# Efficacy and safety of XELOX combined with anlotinib and penpulimab vs XELOX as an adjuvant therapy for ctDNA-positive gastric and gastroesophageal junction adenocarcinoma: a protocol for a randomized, controlled, multicenter phase II clinical trial (EXPLORING study)

**DOI:** 10.3389/fimmu.2023.1232858

**Published:** 2023-10-31

**Authors:** Yizhang Chen, Jiaguang Zhang, Gaohua Han, Jie Tang, Fen Guo, Wei Li, Li Xie, Hao Xu, Xinyi Zhang, Yitong Tian, Lanlan Pan, Yongqian Shu, Ling Ma, Xiaofeng Chen

**Affiliations:** ^1^ Department of Oncology, The First Affiliated Hospital of Nanjing Medical University, Nanjing, China; ^2^ The Affiliated Wuxi People's Hospital of Nanjing Medical University, Wuxi People's Hospital, Wuxi Medical Center, Nanjing Medical University, Wuxi, China; ^3^ Department of Oncology, The Affiliated Taizhou People's Hospital of Nanjing Medical University, Taizhou, China; ^4^ Department of Oncology, Liyang People's Hospital, Changzhou, China; ^5^ Department of Oncology, Suzhou Municipal Hospital, Suzhou, China; ^6^ Department of Oncology, The First Affiliated Hospital of Soochow, Suzhow, China; ^7^ Clinical Research Institute, Shanghai Jiao Tong University School of Medicine, Shanghai, China; ^8^ Department of Gastric Surgery, The First Affiliated Hospital of Nanjing Medical University, Nanjing, China

**Keywords:** clinical trial, GA/GEJA, anlotinib, penpulimab, XELOX adjuvant chemotherapy, CtDNA, vascular normalization, protocol

## Abstract

**Background:**

The efficacy of current adjuvant chemotherapy for gastric adenocarcinoma/gastroesophageal junction adenocarcinoma (GA/GEJA) leaves much to be desired. ctDNA could serve as a potential marker to identify patients who are at higher risk of recurrence. Reinforcing standard adjuvant chemotherapy with immunotherapy has already been indicated to significantly improve clinical outcome, albeit such evidence is rare in GA/GEJA. Here, we intend to explore the clinical benefit of the reinforcement of adjuvant immunotherapy and antiangiogenics alongside with chemotherapy in patients who are deemed in high risk of recurrence by ctDNA analysis, which might shed light on further improvements in adjuvant therapy for GA/GEJA.

**Methods/Design:**

This study is designed as a prospective, multicenter, randomized, controlled phase II study in patients histologically or cytologically diagnosed with GA/GEJA who underwent D2 gastrectomy and achieved R0 or R1 resection. From February 2022, a total of 300 stage III patients will be enrolled and subjected according to ctDNA sequencing results, and those with positive results will subsequently be randomized 1:1 to arm A or B. Patients in arm A will receive anlotinib, penpulimab and XELOX for 6-8 cycles, maintained with anlotinib and penpulimab for up to 1 year, while patients in arm B will receive XELOX alone for 6-8 cycles. ctDNA-negative patients will be assigned to arm C, and patients who are ctDNA positive but failed in randomization will be assigned to arm D. Patients in arms C and D will receive the investigator’s choice of therapy. The primary endpoint is the median disease-free survival (DFS) of arm A versus arm B determined via CT/MRI imaging. Secondary endpoints include the DFS of ctDNA positive patients versus ctDNA negative patients, the 2- and 3-year DFS rates, overall survival (OS), the impact of hallmark molecules on the treatment response, adverse events (AEs), and the impact of nutrition status or exercise on recurrence.

**Discussion:**

We expect that ctDNA would be a strong prognostic factor and ctDNA-positive patients are at higher risk of relapse than ctDNA-negative patients. The addition of anlotinib and penpulimab to XELOX, may contribute to delaying relapse in ctDNA-positive patients.

**Trial registration:**

https://www.clinicaltrials.gov, identifier NCT05494060.

## 1 Background

Gastric cancer (GC) is a malignant tumor occurring in the digestive tract; it ranks fifth in occurrence among all malignancies and third in mortality. Chinese GC patients constitute 47% of patients worldwide (1). There were approximately 478,000 new occurrences and 374,000 fatalities in China in 2020, and the occurrence and fatality of GC rank third among all malignancies in the Chinese population (2). For stage II patients, the recommended regimen is S-1 alone (per oral to 1 year after surgery) or capecitabine plus oxaliplatin/cisplatin (3, 4). In 2022, the 3-year follow-up report of the JACCRO GC-07 trial suggested administering 6 cycles of docetaxel with S-1 (DS regimen) in stage III GC patients which improved the OS of patients compared to single-regimen S-1 (5). In 2019, the RESOLVE trial showed that for cT4aN+M or cT4bNxM locally advanced GC, adjuvant chemotherapy with 8 cycles of the S-1 with oxaliplatin (SOX) regimen is noninferior to the XELOX regimen (6). The 3-year DFS of patients enrolled in these trials is approximately 50% - 85%. However, for stage III patients, the 3-year DFS is approximately 50% - 65%, which implies a significant risk of recurrence in patients receiving standard postoperative adjuvant chemotherapy.

The identification of patients who are most at risk and the precise administration of reinforced therapy are the focus in further improving the perioperative treatment. Circulating tumor DNA (ctDNA) is a biomarker with promising prospects of serving as a prognostic indicator. Kotani et al. reported that postsurgical ctDNA in patients with stage II–IV resectable CRC is found to be associated with higher recurrence risk (hazard ratio (HR) 10.0, *P* < 0.0001). Furthermore, postsurgical ctDNA positivity identified patients with stage II or III CRC who derived benefit from adjuvant chemotherapy (HR 6.59, *P* < 0.0001) (7). In addition, Gale et al. used ctDNA as an indicator for risk of relapse in localized non-small cell lung cancer (NSCLC) following treatment with curative intent. Detection within 2 weeks to 4 months after treatment was associated with shorter recurrence-free survival [hazard ratio (HR): 14.8, *P<*0.00001] and OS (HR: 5.48, *P<*0.0003) (8). In GC, Yang et al. reported a significant correlation of worse DFS and OS. (HR = 14.78, 95%CI, 7.991–61.29, *P* < 0.0001 and HR = 7.664, 95% CI, 2.916–21.06, *P* = 0.002, respectively), and preceded radiographic recurrence by a median of 6 months (9). From these studies we can conclude that positive ctDNA is a sensitive risk indicator for recurrence and calls for reinforced means to mitigate this risk. However, the number of patients participating in studies on the value of ctDNA as a risk indicator of recurrence in postoperative GC patients was rather limited, calling for additional verification. Studies supporting the use of immune checkpoint inhibitors (ICIs) in the adjuvant setting in gastroesophageal cancer are also rare. The results of CheckMate-577 revealed that disease-free survival (DFS) was significantly longer in nivolumab group than in placebo group in GA/GEJA patients (HR for disease recurrence or death, 0.69; 96.4% CI, 0.56 to 0.86; *P<*0.001) (10). Similar researches also suggest a benefit in ICIs adjuvant therapy, such as the CheckMate-274 trial in urothelial cancer (11) and the MAGE-A3 trial in NSCLC (12). In summary, the value of ctDNA as a risk factor and the clinical benefit of ICIs as a part of adjuvant therapy requires further evidence.

Therefore, we designed this clinical trial, providing ctDNA screening to stage III GC patients who underwent curative gastrectomy, and randomized them into different arms based on ctDNA results. The trial arm will be treated with chemotherapy combined with ICIs and antiangiogenics. The control arm will be given standard chemotherapy. We hope to further explore the prognostic value of ctDNA and whether reinforced adjuvant treatment for ctDNA-positive patients can improve the prognosis.

## 2 Patients and methods

### 2.1 Recruitment and allocation

Patients with pathologically and/or cytologically diagnosed GA/GEJA who underwent D2 gastrectomy were potentially eligible for enrollment, and every consenting patient signed the informed consent form (ICF) prior to actual enrollment.

The criteria for eligible patients included 18- to 75-year-old males and females (female participants should not be pregnant or lactating), patients histologically or cytologically diagnosed with stage III GA/GEJA (Siewert III), patients who underwent D2 gastrectomy and achieved R0 or R1 resection, patients with an Eastern Cooperative Oncology Group (ECOG) performance status between 0 to 1, and patients without significant defects in organ function. Considering that the detection of ctDNA via next-generation sequencing (NGS) may take up to 10 working days, patients who received only the first cycle of XELOX chemotherapy will also be accepted for enrollment. The major exclusion criteria included serious/uncontrollable hypertension/cardiac insufficiency/infection/diabetes/renal insufficiency, tendency for gastrointestinal hemorrhage, immune deficiency, recent thrombotic event or any other situation deemed unsuitable for enrollment by the researcher. A more detailed version of our inclusion/exclusion criteria is provided in **Supplementary Materials**.

The recruited patients will be subjected to ctDNA analysis (Geneseeq Technology Co., Ltd., Nanjing, China; 1021 genes NGS) 3-8 weeks after the operation. Patients with positive ctDNA will be randomly allocated to two arms (arms A&B). Patients with negative ctDNA will constitute arm C. Patients who are ctDNA positive but for various reasons fail in random allocation will be assigned to arm D.

Block randomization is used in this study, with a block size of 4. Random allocation sequence including randomization seed, block size and block counts are provided by our statistics specialist. A central randomization is established, in which researchers will enter the participants’ general information, including abbreviation of participant’s name, gender and age to apply for a randomization number and allocation into arms. This randomization number will be included in the participant’s medical history, and the participant will receive treatment according to the allocated arm.

### 2.2 Trial design

This study was designed as a prospective, multicenter, randomized, controlled, open-label and multicenter phase II clinical trial conducted in China. beginning in June 2022. Patients in arm A (experiment group) will undergo XELOX chemotherapy combined with anlotinib and penpulimab until they reach the 6-8 cycle without recurrence. Subsequently, they will receive anlotinib combined with penpulimab as maintenance therapy until recurrence or death occurs or reach the maximum length of 1 year. Patients in arm B (control group) will undergo XELOX chemotherapy for 6-8 cycles. Arm C&D will receive the researcher’s choice of treatment according to recommendations by the Chinese Society of Clinical Oncology (CSCO) guidelines. All ctDNA positive patients should receive ctDNA tests every 3 months as a dynamic monitoring procedure. Considering that NGS analysis may take up to 10 working days, patients could receive 1 cycle of XELOX chemotherapy after ctDNA sampling. Follow-ups and evaluations will be carried out regularly. In addition to data concerning treatment and recurrence, data on nutritional and exercise habits will also be collected in an exploratory attempt to determine the impact of nutrition and exercise on survival and the risk of recurrence.

The primary endpoint of our trial is the DFS of ctDNA-positive patients receiving XELOX combined with anlotinib and penpulimab versus ctDNA-positive patients receiving XELOX alone (arm A versus arm B). The secondary endpoints include the DFS of ctDNA-positive patients versus ctDNA-negative patients, the 2-year DFS rate, the 3-year DFS rate, overall survival (OS), the relationship of dynamic ctDNA status and treatment response, adverse events (AEs), and the impact of nutrition status and exercise on recurrence.

### 2.3 ctDNA sequencing via NGS

ctDNA from the plasma was extracted using a QIAmp Circulating Nucleic Acid Kit (Qiagen). Genomic DNA from surgical tumor samples and white blood cells was extracted using a DNeasy Blood & Tissue kit (Qiagen). Purified genomic DNA was qualified by Nanodrop2000 for A260/280 and A260/A230 ratios (Thermo Fisher Scientific). All DNA samples were quantified by Qubit 3.0 using the dsDNA HS Assay Kit (Life Technologies) according to the manufacturer’s recommendations. Hereafter, a local bioinformatics polishing pipeline was used to identify somatic variants in ctDNA after filtering out germline variants using normal control DNA. Mutations identified in the matched tumor DNA, which were supported by a minimum of one unique consensus mutant allele read and passed the polishing criteria, were regarded as being present.

### 2.4 Intervention and assessment

Arm A (experimental group) will be given anlotinib 12 mg, po, qd + penpulimab 200 mg, iv, d1 + XELOX regimen (oxaliplatin 130 mg/m^2^, iv, d1 + capecitabine 1000 mg/m^2^, po, qd, d1-14). Arm B will be given the same XELOX regimen as arm A. Arms C&D will be given the investigator’s choice of treatment. If drug-associated toxicity occurs during treatment, it is up to the investigator to consider the effect of the toxicity (according to the National Cancer Institute (NCI) Common Terminology Criteria for Adverse Events (CTCAE 5.1) and the therapeutic benefit and decide whether to adjust the dose of anlotinib or penpulimab. The starting dose of anlotinib is 12 mg qd and can be decreased to 10 mg or 8 mg. A dose reduction or suspension is allowed only when ≥ grade 3 hematological toxicity or ≥ grade 2 nonhematological toxicity is observed. Regarding penpulimab, adjustment of the dose is not recommended. Management of the AEs of penpulimab should be in accordance with the CSCO guidelines for the management of immune checkpoint inhibitor-related toxicity. If the administration of penpulimab is postponed for more than 12 weeks due to infusion-related AEs or more than 3 weeks due to immune-related AEs, the usage of penpulimab should be permanently discontinued. The study flowchart is shown in **Supplementary Materials Figure 1**. Supportive treatment including antibiotics, pain reliefs, steroids, palliative surgery is considered acceptable as a concomitant care. Any other anti-tumor chemo/endocrine/immune treatment is considered not acceptable.

The results of follow-up examinations should be closely monitored (see **Supplementary Materials** for details), including ctDNA status every 3 months (in ctDNA-positive patients only) and nutrition status, in a case report form (CRF). Imaging via CT or MRI will be performed before the first administration of drugs, after every 2 cycles and upon treatment completion. Upon and after treatment completion, patients will still be regularly followed-up for survival.

### 2.5 Data collection and management

After obtaining ICFs signed by the patients, our clinical investigators will begin collecting the baseline data, including age, gender, body mass index and adverse events, along with all laboratory indices and imaging that are required (refer to “Intervention and Assessment” & **Supplementary Materials** for further details). Peripheral blood and tissue samples will be collected for cytometry analysis, immune environment analysis and genetic sequencing.

All clinical data relevant to our patients will be recorded in CRFs on a regular basis by trained and independent clinical research coordinators (CRCs) and managed by a data manager based at the main center. All data will be verified by the data manager and the principal investigator. The CRFs will be under constant surveillance by the clinical investigators to minimize the possibility that any mistake remains unchecked. All research data will be available exclusively to researchers who have signed a confidential disclosure agreement. In particular, any collected information in this study that may potentially lead to the disclosure of any patient’s identity will not be released until consented by the patient, unless special urgent or legal circumferences dictate otherwise. Should any potential error or missing in the data be identified, the rectification will be made only under the approval of the principal investigator, collaborator, statistician and data manager. The ethics committees of the First Affiliated Hospital of Nanjing Medical University will enforce the rights and welfare of all the patients, adherence to the approved version of the protocol, proper practices in collecting clinical data and performing statistical analysis, and anonymity in publications.

### 2.6 Statistical analysis

Previous research reported that the median DFS of postoperative adjuvant chemotherapy in ctDNA-positive GA/GEJA patients is approximately 12 months (13, 14). Our hypothesis is that anlotinib plus penpulimab combined with XELOX will prolong DFS to 24 months. Setting the single-sided α as 0.05, β as 0.2, the length of in-group time as 18 months and the length of follow-up as 24 months, the calculated sample size is 36 patients for the test group (arm A) and 36 patients for the control group (arm B); presuming that the drop-off rate is 10%, the total number of participants needed is 40 each group. The key secondary endpoint is the difference in DFS between ctDNA-positive and ctDNA-negative patients treated by only chemotherapy. Given that the median DFS of ctDNA-negative patients receiving standard treatment is approximately 36 months, two-sided α = 0.05, β = 0.2, the length of in-group time is 18 months, and the length of follow-up is 36 months, the sample size should be 14 each for the ctDNA-positive group and the ctDNA-negative group. Taking both hypotheses into consideration, we presume at least 80 ctDNA-positive patients would be needed to complete this study. Considering that the positive rate of ctDNA in postoperative GA/GEJA patients is approximately 25-30%, a total of 300 participants needs to be screened.

Statistical analysis will be carried out by statistics specialists who will take part in the study during the whole process, from the study design and implementation to the data analysis phase. Measurement variables will be analyzed by means of *t test*, paired *t test*, rank-sum test, paired rank-sum test, analysis of variance (ANOVA), etc. Count variables will be analyzed using the Cochran–Mantel–Haenszel (CMH) chi-squared test, logistic regression and Fisher’s exact test. Ordinal variables will be analyzed via the rank-sum test. Survival data will be compared via the Kaplan–Meier method or Cox regression. The main clinical response indicators will be subjected to per-protocol population (PP) and full analysis set (FAS) analyses simultaneously. A two-sided test for significance was performed as the default, and *P<*0.05 was considered significant. The analysis will be performed using SAS software. The data analysis is planned to be at mid-term and upon completion of this trial.

## 3 Discussion

GC is still a major health threat to China, with over half of the world’s incidences occurring in this country (15). The prognosis of GC patients in China is also less optimistic than that of patients in Europe or the US (16). Currently, a multimodal approach comprising surgery, chemotherapy, targeted therapy, and immunotherapy is employed in the management of GC. Among them, surgery is considered the foundation of our strategy in pursuit of complete remission. However, quite a few patients experience recurrence early, even if they undergo curative surgery and adjuvant chemotherapy (17–19). Therefore, the reinforcement of adjuvant immunotherapy for patients with high recurrence probability is necessary.

Adjuvant immunotherapy has been reported to provide clinical benefits. Lee et al. conducted a trial on post-operation hepatocellular cancer patients. The median time of recurrence-free survival was 44.0 months in the immunotherapy group and 30.0 months in the no-adjuvant control group (*P=*0.010) (20). KEYNOTE-716 trial showed that pembrolizumab as adjuvant therapy for up to approximately 1 year for resected stage IIB or IIC melanoma. Compared with placebo, pembrolizumab as adjuvant therapy resulted in a significant reduction in the risk of disease recurrence or death. In the first interim analysis, 54 (11%) of 487 patients in the pembrolizumab group and 82 (17%) of 489 in the placebo group had a first recurrence of disease or died (hazard ratio [HR] 0.65 [95% CI 0.46-0.92]; *P=*0.0066) (21). Thus, adjuvant immunotherapy plays a significant role in improving clinical benefits of patients undergoing postoperative adjuvant treatment.

In ctDNA positive urothelial carcinoma patients, Powles et al. reported an improved DFS and OS in the atezolizumab arm versus the observation arm (22). Therefore, ctDNA positive patients are most probably to benefit from adjuvant immunotherapy. The ATTRACTION-5 study revealed that N+C (Nivolumab plus chemotherapy) vs P+C (placebo plus chemotherapy) in patients with pathological stage III G/GEJ cancer after D2 or more extended gastrectomy did not meet the primary endpoint of RFS. However, benefit of survival was observed in stage IIIc *GC* patients with higher risk of recurrence. The results of these research suggest that post-operation patients that are in increased risk of recurrence, such as later stage and ctDNA positive status, may benefit from reinforcement of immunotherapy.

ctDNA is one of the means to screen out high-risk patients. Postsurgical ctDNA in patients with stage II–IV resectable CRC is found to be associated with higher recurrence risk. In addition, postsurgical ctDNA identified patients with stage II or III CRC who derived benefit from adjuvant chemotherapy (7). In GC, Yang et al. reported a significant correlation of worse DFS/OS and positive ctDNA with a preceded radiographic recurrence by a median of 6 months (9). Thus, we hypothesized that ctDNA sequencing can screen out high-risk post-operation GC patients and reinforce their adjuvant regimen with immunotherapy might yield positive results.

Approximately a number of 10^6^ tumor cells can be detected in ctDNA positive patients (23). While for imaging examination like CT, a number of 10^12^ tumor cells or an 8 mm diameter lesion size can be detected. Thus, ctDNA sequencing can be used to find earlier tumor lesion than imaging examination. Besides, the process of angiogenesis starts as early as the tumor reached the size of 2 mm (24, 25). At the same time, early intervention with antiangiogenics might be more effective.

Prior to this study, we have already conducted two study in advanced GC with similar regimens: SPACE trial (apatinib, camrelizumab and SOX regimen, ChiCTR2000034109) and TALENT trial (Anlotinib, tislelizumab and XELOX regimen, NCT04963088). Both yields promising results. In the SPACE trial, the confirmed ORR was 80.8%, median PFS was 10.2 months (95% CI, 5.5-22.3), with the median OS not reached yet (26). In the TALENT trial, The ORR was 75.86% (95% CI: 57.89%-87.78%), and the DCR was 100% (27). Considering the fact that stage III patients are generally in better condition than those with advanced cancer, we expect this regimen can not only reduce risk of recurrence, but also have tolerable adverse effects. Therefore, it is necessary to reinforce adjuvant immunotherapy and antiangiogenics in ctDNA positive GA/GEJA patients undergoing curative surgery and adjuvant chemotherapy.

Our study aims to explore the potential of ctDNA as a biomarker to screen out the patients that are most at risk for recurrence, and the efficacy and safety of reinforcing adjuvant chemotherapy with targeted as well as immunotherapy in high-risk patients determined by ctDNA status. Both of them are novel to adjuvant treatment of *GC*. This study is also designed to have multiomics analysis, including genomics, transcriptomics, microenvironment immune cell infiltration, radiomics and nutritional analysis, which will help not only to validate the use of ctDNA as an immunotherapy indicator, but also to explore the mechanics that lies beneath. We are the first to report the use of ctDNA as an indicator of reinforced adjuvant therapy, in stage III GA/GEJA. Our data can provide the much needed and long-awaited evidence to support clinical decision in implementing adjuvant immunotherapy in GA/GEJA. However, some limitations remain. Firstly, as a researcher-initiated study, the scale of our study is limited. Expanded trials featuring more patients would provide valuable insight into specific findings derived from subgroups. Secondly, dynamic monitoring of ctDNA status is not applied to all our patients, which means conversion of ctDNA negative patients could be neglected, thus impacts of therapy in this specific population remains unclear. Lastly, only stage III patients, no high-risk stage II patient is enrolled in our study. As early recurrence is also seen in stage II patients, the portion of ctDNA positive patients, prognostic value of ctDNA status and clinical benefit of radical adjuvant therapy cannot be explored in this study. We hope our study can provide novel insights into the clinical use of ctDNA and help to further develop the paradigm of precision medicine.

## 4 Trial status

We estimate that this clinical study will last for 18 months, starting from July 2022 to February 2024. Patients will be recruited from multiple centers across Jiangsu Province. A total of 300 patients will be screened for this trial.

## Ethics statement

The studies involving humans were approved by the ethics committee of the First Affiliated Hospital of Nanjing Medical University. The studies were conducted in accordance with the local legislation and institutional requirements. The participants provided their written informed consent to participate in this study.

## Author contributions

All authors contributed to the conception, data analysis, and drafting of the article and have independently reviewed this version of the manuscript, giving their approval for submission to this journal. All authors agree to be held accountable for all aspects of this work. All authors contributed to the article and approved the submitted version.
