# Supplementary material for: Efficacy and safety of XELOX combined with anlotinib and penpulimab vs XELOX as an adjuvant therapy for ctDNA-positive gastric and gastroesophageal junction adenocarcinoma: a protocol for a randomized, controlled, multicenter phase II clinical trial (EXPLORING study)
Source: Front Immunol. 2023 Oct 31;14:1232858. doi: 10.3389/fimmu.2023.1232858 (PMC10644233; doi:10.3389/fimmu.2023.1232858)
Supplement: Supplementary file 1 [file DataSheet_1.docx]

***Supplementary Material***

**Efficacy and safety of XELOX combined with anlotinib and penpulimab vs XELOX as an adjuvant therapy for ctDNApositive gastric and gastroesophageal junction adenocarcinoma: a protocol for a randomized, controlled, multicenter phase II clinical trial (EXPLORING study)**

Yizhang Chen^1,2*^, Jiaguang Zhang^1*^, Gaohua Han^3*^, Jie Tang^4^, Fen Guo^5^, Wei Li^6^, Li Xie^7^, Hao Xu^8^, Xinyi Zhang^1^, Yitong Tian^1^, Lanlan Pan^1^, Yongqian Shu^1^, Ling Ma^1#^, Xiaofeng Chen^1,3#^

^1^: Department of Oncology, The First Affiliated Hospital of Nanjing Medical University, Nanjing, China

^2^: The Affiliated Wuxi People's Hospital of Nanjing Medical University, Wuxi People's Hospital, Wuxi Medical Center, Nanjing Medical University, Wuxi, China

^3^: Department of Oncology, The Affiliated Taizhou People's Hospital of Nanjing Medical University, Taizhou, China

^4^: Department of Oncology, Liyang People's Hospital, Changzhou, China

^5^: Department of Oncology, Suzhou Municipal Hospital, Suzhou, China

^6^: Department of Oncology, The First Affiliated Hospital of Soochow, Suzhow, China

^7^: Clinical Research Institute, Shanghai Jiao Tong University School of Medicine, Shanghai 200025, China.

^8^: Department of Gastric Surgery, the First Affiliated Hospital of Nanjing Medical University, Nanjing, China

*Yizhang Chen, Jiaguang Zhang and Gaohua Han contributed equally to this work.

**#Corresponding author:**

Ling Ma

Department of Oncology, The First Affiliated Hospital of Nanjing Medical University, No. 300,

Guangzhou Road, Nanjing, Jiangsu Province, China, 210029

maling@njmu.edu.cn

Xiaofeng Chen

Department of oncology, the First Affiliated Hospital of Nanjing Medical University, No. 300,

Guangzhou Road, Nanjing, Jiangsu Province, China, 210029

chenxiaofengnjmu@163.com

# Inclusion and Exclusion Criteria

Inclusion Criteria:

1. The subject’s age must be between 18 and 75. Both males and females are eligible.
2. Subjects must have stage II-III histopathologically/cytologically diagnosed gastric adenocarcinoma/gastroesophageal junction adenocarcinoma (GA/GEJA, Siewert III) and have undergone D2 gastrectomy, reaching R0 or R1 resection.
3. The subject’s Eastern Cooperative Oncology Group performance score (ECOG PS) falls between 0 and 1.
4. The expected length of survival was no less than 6 months.
5. Subjects must have sufficient heart function, with no episode of cardiac infarction within half a year prior to enrollment. If the subject has coronary heart disease, the condition should be manageable.
6. Sufficient function of vital organs, as follows:
   1. Routine blood tests should satisfy these criteria:
      1. The concentration of hemoglobin (HB) should be no less than 90 g/L, without receiving a blood infusion 28 days prior to enrollment.
      2. The absolute neutrophil count (ANC) should be no less than 1.5x10^9^/L.
      3. The platelet count (PLT) should be no less than 100x10^9^/L.
   2. The biochemistry analysis should satisfy these criteria:
      1. Serum total bilirubin (TBIL) should not surpass 1.5 times the upper limit of normal (ULN).
      2. Alanine aminotransferase (ALT) and aspartate aminotransferase (AST) should not surpass 2.5 times the ULN.
      3. Serum creatine (Cr) should not surpass 1.5 times the ULN, or creatine clearance (CCr) should be no less than 60 ml/min (estimated via Cockcroft-Gault equation).
   3. Sufficient coagulation function, defined by the international normalized ratio (INR) and prothrombin time (PT), should not surpass 1.5 times the ULN.
7. Subjects should be without other uncontrollable diseases in the lungs, kidney or liver or infections, even though they are benign.
8. Considering that NGS analysis may take up to 10 working days, patients could receive 1 cycle of XELOX chemotherapy after ctDNA sampling.
9. Patients must agree to participate and sign our informed consent form (ICF).
10. Female subjects of reproductive age should take appropriate means of contraception and avoid breastfeeding from enrollment to 3 months after the termination of research treatments. The subject must undergo a pregnancy test before treatment, or one of the following criteria must be met as proof of no risk of pregnancy:
    1. Postmenopausal, defined as older than 50 years of age or at least 12 months of amenorrhea after the discontinuation of all external hormone substitution therapy.
    2. Female subjects below 50 years of age who had amenorrhea for at least 12 months after the discontinuation of all external hormone substitution therapy and whose levels of luteinizing hormone (LH) and follicle stimulating hormone (FSH) were within the referential value of menopausal females were considered menopausal.
    3. Subjects who received irreversible sterilization procedures, including uterine resection, bilateral ovarian resection, or bilateral fallopian tube resection, were considered sterile. However, patients who undergo bilateral fallopian tube ligation are not considered sterile.

Male subjects, on the other hand, should give consent to take proper means of contraception in the duration of this study and 8 weeks after the last administration of trial drugs or have received sterilization procedures.

Exclusion Criteria:

1. Subjects with any severe or uncontrolled diseases, including:
   1. Hypertension that cannot be satisfactorily controlled by a single drug regimen (systolic pressure ≥ 140 mmHg, diastolic pressure ≥ 90 mmHg) or the administration of multiple drugs simultaneously is needed to control the hypertension.
   2. Acute myocardial infarction, malign arrythmia (including Q-T interval > 450 ms in males, or Q-T interval > 470 ms in females), class II and above congestive heart failure, according to the New York Heart Association (NYHA) criteria, or echocardiogram reports a left ventricular ejaculation fraction (LVEF) below 50%.
   3. Active or uncontrolled infection, in accordance with grade 2 infections defined by the National Cancer Institute’s Common Terminology Criteria for Adverse Events (NCI-CTC AE).
   4. Diabetes mellitus (DM) that was not satisfactorily controlled was defined as fasting blood glucose (FBG) > 10 mmol/L.
   5. Urine routine report urine protein ≥ 2+, validated by quantified 24-h urine protein > 1.0 g.
2. Major bleeding is deemed likely by the investigator.
3. Insufficient coagulation functions, defined as prothrombin time (PT) > 16 s, activated partial prothrombin time (APTT) > 43 s, thrombin time (TT) >21 s, fibrinogen (Fbg) <2 g/L, or tendency of hemorrhage, whether due to thrombolysis/anti-coagulation treatment.
4. Unable to swallow, chronic diarrhea, intestinal obstruction, or any other issue that denies administering drugs orally.
5. A previous history of severe autoimmune diseases, including but not limited to autoimmune hepatitis, myasthenia gravis, systemic lupus erythema, etc. Patients with moderate and well-controlled autoimmune diseases, such as leukoderma or rash, can be admitted.
6. Previous history of immune deficiency, including HIV positivity, any other congenital or acquired immune disease, or recipient of an organ transplantation.
7. Thrombotic events in the previous 6 months, such as cerebrovascular events (including transient ischemic attacks), deep vein thrombosis or pulmonary embolism.
8. Subjects who have a history of psychoactive drug abuse or suffer from mental health issues.
9. The subject had confirmed allergies to anlotinib, penpulimab or any excipients.
10. The subject have recieved neoadjuvant therapies.
11. Subjects who have other diseases that pose grave danger to the patient or interfere with the treatment according to the investigator’s judgment.
12. Any other issues deemed unfit for enrollment by the investigator.

Note: patients excluded according to the first three exclusion criteria will be included in the safety analysis.

Criteria for Discontinuation of Treatment

1. Trial participant or his/her legal representatives demand withdrawal from the study.
2. Continuation of the study is deemed inappropriate due to medical or ethical concerns.
3. The researchers decided that withdrawal from the study is in the participant’s best interest.
4. The participant was lost to follow-up.
5. The participant was pregnant
6. Serious deviation from the trial protocol (including intolerable deviation from the inclusion criteria).

# Supplementary Figures


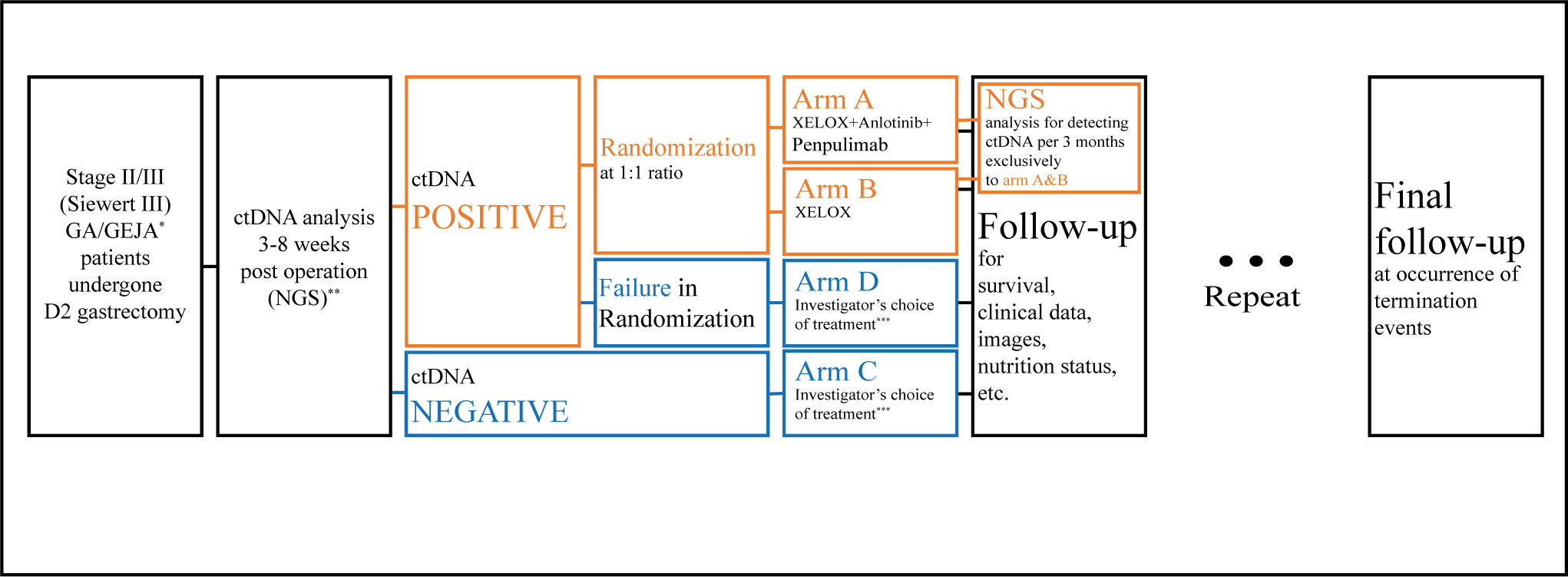


**Supplementary Figure 1.** A brief outline of the protocol of this study.

*: Gastric adenocarcinoma/gastroesophageal junction adenocarcinoma.

**: Next-generation sequencing.

***: Investigator’s choice based on the CSCO guidelines for gastric cancer, including single regimen S-1, XELOX, SOX, XP, sequential DS/S-1 and FOLFOX. For patients who did not reach D2 lymphotomy or who underwent R1 resection, postoperative chemoradiotherapy was also an option.

# Data metrics

# Before enrollment

1. Medical history and general information, including ID number, gender, age, corresponding address and phone number.
2. History of treatment, physical examination, ECOG PS, height, weight, vital signs.
3. Radiology examination results (CT/MRI within 4 weeks), thyroid gland function, 12-lead electrocardiogram (ECG) with Q-T interval (QTc) recorded, echocardiogram, and myocardial zymography.
4. Blood, urine, and feces routine tests including occult blood (OB) in feces.
5. Alanine aminotransferase (ALT), aspartate aminotransferase (AST), glutamyl transpeptidase (GGT), total bilirubin (TBIL), direct bilirubin (DBIL), alkaline phosphatase (ALP), blood urea nitrogen (BUN), creatine (Cr), blood glucose (GLU), potassium ion (K+), sodium ion (Na+), calcium ion (Ca2+), chloride ion (Cl-) in blood. Female participants that are of childbearing age should receive human chorionic gonadotropin (HCG) test to rule out pregnancy.
6. Coagulation function test (prothrombin time (PT), active partial thromboplastin time (APTT), thrombin time (TT), fibrinogen (Fbg), international normalization ratio (INR)), tumor indicators (carcinoembryonic antigen (CEA), alpha fetoprotein (AFP), carbohydrate antigen 199 (CA199), carbohydrate antigen 724 (CA724)) should be examined every cycle.
7. Hepatitis B antibody and antigen, hepatitis C and human immunodeficiency virus (HIV) related blood tests.
8. ctDNA test 3-8 weeks post operation before initiation of adjuvant therapy.
9. Blood, feces sample and 25 unstained pathological slides of the surgical resected tissue for further exploring molecule markers and genetic sequencing.
10. Diet, exercise, and quality of life assessment, collected via questionnaire by clinical investigator.
11. Other tests deemed necessary by the clinical investigator.

# During study

1. Blood pressure measured at least 3 times a day during drug administration.
2. Physical examinations of general condition (ECOG PS), skin & mucosa, head, neck, chest, abdomen, spine & limbs and vital signs other than blood pressure (body temperature (T), pulse rate (P) and respiration rate (R)) should be recorded every cycle.
3. Any adverse effects during treatment, including nausea, vomiting, diarrhea, abdominal distension etc. should be recorded.
4. Any concomitant care received during treatment should be recorded.
5. Coagulation function test (PT, APTT, TT, INR) should be examined every cycle.
6. Blood routines including erythrocyte count (RBC), hemoglobin (Hb), leukocyte count (WBC), neutrophils count (ANC), lymphocyte count (LYM) examined per cycle during the first 4 cycles, then examined every 2 cycles. If ANC falls beneath 1×10^9^/L or platelet (PLT) falls beneath 50×10^9^/L, the blood routines should be examined every 2-3 days. Should postpone of administer or dosage change occurred due to hematological toxicity, blood routines should be examined every cycle.
7. Urine routines including urine protein, urine glucose, urine occult blood (urine erythrocyte count or urine hemoglobin), urine acidity and urine ketone should be examined every cycle. If half-quantified tests (i.e., test strips)reports urine protein is greater than 2+, quantified 24h urine protein should be examined.
8. Feces routines including occult blood should be examined every cycle. This can be omitted if samples of feces is not available due to conditions like constipation.
9. Serum biochemical analysis including ALT, AST, GGT, TBIL, DBIL, ALP, BUN, total protein (TP), albumin (ALB), Cr, GLU, K+, Na+, Ca2+, Cl- examined every cycle.
10. Tumor indicators including but not limited to CEA, AFP, CA199, CA724 should be examined every cycle.
11. 12-lead ECG recorded every cycle. If the ECG record was abnormal, 2 additional ECG records with QTc labeled should be made at the interval of 5 minutes each as conformation.
12. Myocardial zymography should be examined every 2 cycles.
13. Thyroid gland functions including thyroid-stimulating hormone (TSH), free triiodothyronine (FT3), free tetraiodothyronine (FT4) should be examined every 2 cycles.
14. Radiology examinations should be made at the end of every 2 cycles, or whenever a new tumor focus is suspected. Conditions of the scan (i.e., scanning layer thickness, contrast agent, etc.) should be identical to the baseline scan. The timeframe allowed for the scan is ±7 days from last day of treatment cycle.
15. ctDNA analysis for participants allocated to arm A & B.
16. Diet, exercise, and quality of life assessment, collected via questionnaire by clinical investigator.
17. Other tests deemed necessary by the clinical investigator.

# After completion/termination of treatment

Safety follow-up is performed after 30±7 days upon completion of last treatment or termination of treatment. If the participant is to receive further line of therapy, then this follow-up should be completed before the initiation of further line treatment.

1. Blood pressure measured at least 3 times a day.
2. Physical examinations of general condition (ECOG PS), skin & mucosa, head, neck, chest, abdomen, spine & limbs and vital signs other than blood pressure (body temperature (T), pulse rate (P) and respiration rate (R)).
3. Any adverse effects during treatment, including nausea, vomiting, diarrhea, abdominal distension etc. should be recorded until all adverse effects relieved to NCI-CTCAE class 1 or resided.
4. Tumor indicators including but not limited to CEA, AFP, CA199, CA724.
5. Coagulation function test (PT, APTT, TT, INR).
6. Blood routines including RBC, Hb, WBC, ANC, LYM.
7. Urine routines including urine protein, urine glucose, urine occult blood (urine erythrocyte count or urine hemoglobin), urine acidity and urine ketone.
8. Feces routines including occult blood. This can be omitted if samples of feces is not available due to conditions like constipation.
9. Serum biochemical analysis including ALT, AST, GGT, TBIL, DBIL, ALP, BUN, TP, ALB, Cr, GLU, K+, Na+, Ca2+, Cl-.
10. 12-lead ECG.
11. Myocardial zymography.
12. Thyroid gland functions TSH, FT3, FT4.
13. Radiology examinations should be made every 6 months, until relapse or initiation of further line of treatment. After relapse, the radiology scan is no longer necessary and only the survival status and subsequent treatment is recorded. If the participant died, the date of death should be recorded.

# Ethics statement

| Registration number： | NCT05494060 |
| --- | --- |
| Date of Last Refreshed on： | 2022-08-09 |
| Version of Protocol | V1.1 |
| Date of Registration： | 2022-08-09 |
| Registration Status： | Prospective registration |
| Public title： | Efficacy and Safety of XELOX combined with anlotinib and penpulimab vs XELOX as an adjuvant therapy for ctDNA-positive gastric and esophagogastric junction adenocarcinoma: A Protocol for a Randomized, Controlled, Multicenter Phase II Clinical Trial (EXPLORING study) |
| Scientific title： | Efficacy and Safety of XELOX combined with anlotinib and penpulimab vs XELOX as an adjuvant therapy for ctDNA-positive gastric and esophagogastric junction adenocarcinoma: A Protocol for a Randomized, Controlled, Multicenter Phase II Clinical Trial (EXPLORING study) |

| Applicant： | Xiao-Feng Chen | Study leader： | Yong-Qian Shu |
| --- | --- | --- | --- |
| Applicant telephone： | +86 13585172066 | Study leader's telephone： | +86 2568306428 |
| Applicant E-mail： | xiaofengch198019@126.com | Study leader's E-mail： | shuyongqian@csco.org.cn |
| Applicant address： | 300 Guangzhou Road, Nanjing, Jiangsu, China | Study leader's address： | 300 Guangzhou Road, Nanjing, Jiangsu, China |
| Applicant postcode： | 210000 | Study leader's postcode： | 210029 |
| Applicant's institution： | Jiangsu Province Hospital, the First Affiliated Hospital with Nanjing Medical University. | | |

| Approved by ethic committee： | Yes |
| --- | --- |
| Approved No. of ethic committee： | 2022-SR-342 |
| Name of the ethic committee： | Ethics Committee of Jiangsu Province Hospital, the First Affiliated Hospital with Nanjing Medical University. |
| Date of approved by ethic committee： | 2022-06-07 |
| Contact Name of the ethic committee： | Xu Huang |
| Contact Address of the ethic committee： | Floor 7, Pharmacology building (building #7), 300 Guangzhou Street, Gulou District, Nanjing, Jiangsu, China |

| Contact phone of the ethic committee： | +86 25 68306360 |
| --- | --- |
| Primary sponsor： | The First Affiliated Hospital of Nanjing Medical University |
| Primary sponsor's address： | 300 Guangzhou Street, Gulou District, Nanjing, Jiangsu, China |
| Secondary sponsor： | \| Country： \| China \| Province： \| Jiangsu \| City：Nanjing \| Nanjing \| \| --- \| --- \| --- \| --- \| --- \| --- \| \| Institution hospital： \| The Second Affiliated Hospital of Nanjing Medical University \| Address： \| No.121 Jiangjiayuan, Gulou District, Nanjing, Jiangsu, China. \| \|  \| \| Institution hospital： \| Suzhou Municipal Hospital \| Address： \| No.26 Daoqian Street, Gusu District, Suzhou, Jiangsu, China. \| \|  \| \| Institution hospital： \| Affiliated Hospital of Jiangnan University \| Address： \| No.200 Huihe Street, Binhu District, Wuxi, Jiangsu, China. \| \|  \| \| Institution hospital： \| Xuzhou Central Hospital \| Address： \| No.199 South Jiefang Street, Quanshan District, Xuzhou, Jiangsu, China. \| \|  \| \| Institution hospital： \| Wuxi People’s Hospital \| Address： \| No.299 Qingyang street, Liangxi District, Wuxi, Jiangsu, China. \| \|  \| \| Institution hospital： \| Affiliated Hospital of Jiangsu University \| Address： \| No.483 Jiefang Street, Jinkou District, Zhengjiang, Jiangsu, China. \| \|  \| \| Institution hospital： \| Huai’an First People’s Hospital \| Address： \| No.6 West Beijing Street, Huaiyin District, Huaian, Jiangsu, China. \| \|  \| |
| Source(s) of funding： | None |
| Target disease： | Gastric cancer |
| Study type： | Interventional study |
| Study phase： | 2 |
| Study design： | Single arm |

| Study execute time： | From 2022-07 To 2027-02 | Recruiting time： | From 2022-07  To 2024-02 |
| --- | --- | --- | --- |

| Interventions： | \| Group： \| Random controlled \| Sample size： \| 80 \| \| --- \| --- \| --- \| --- \| \| Intervention： \| Low-dose XELOX combined with anlotinib and penpulimab \| Intervention code： \|  \| |
| --- | --- | --- | --- | --- | --- | --- | --- | --- | --- |

| Countries of recruitment and research settings： | \| Country： \| China \| Province： \| Jiangsu \| City： \| Nanjing \| \| --- \| --- \| --- \| --- \| --- \| --- \| \| Institution hospital： \| The First Affiliated Hospital of Nanjing Medical University/Pukou Branch Hospital of Jiangsu Province Hospital \| Level of the institution： \| Tertiary A \| \| \| \| Institution hospital： \| The Second Affiliated Hospital of Nanjing Medical University \| Level of the institution： \| Tertiary A \| \| \| \| Institution hospital： \| Suzhou Municipal Hospital \| Level of the institution： \| Tertiary A \| \| \| \| Institution hospital： \| Affiliated Hospital of Jiangnan University \| Level of the institution： \| Tertiary A \| \| \| \| Institution hospital： \| Xuzhou Central Hospital \| Level of the institution： \| Tertiary A \| \| \| \| Institution hospital： \| Wuxi People’s Hospital \| Level of the institution： \| Tertiary A \| \| \| \| Institution hospital： \| Affiliated Hospital of Jiangsu University \| Level of the institution： \| Tertiary A \| \| \| \| Institution hospital： \| Huai’an First People’s Hospital \| Level of the institution： \| Tertiary A \| \| \| |
| --- | --- | --- | --- | --- | --- | --- | --- | --- | --- | --- | --- | --- | --- | --- | --- | --- | --- | --- | --- | --- | --- | --- | --- | --- | --- | --- | --- | --- | --- | --- | --- | --- | --- | --- | --- | --- | --- | --- | --- | --- | --- | --- | --- | --- | --- | --- | --- | --- | --- | --- | --- | --- | --- | --- | --- |

| Recruiting status： | Recruiting | Participant age： | \| Min age \| 18 \| years \| \| --- \| --- \| --- \| \| Max age \| 75 \| years \| |
| --- | --- | --- | --- | --- | --- | --- | --- | --- | --- |
|  |  | Gender： | Both |
| Randomization Procedure (please state who generates the random number sequence and by what method)： | Block randomization via a central electronic randomization system | | |

| Blinding： | N/A |
| --- | --- |

| The time of sharing IPD： | Undecided |
| --- | --- |
| The way of sharing IPD”(include metadata and protocol, If use web-based public database, please provide the url)： | Date with 6 months after the trial complete |
| Data collection and Management (A standard data collection and management system include a CRF and an electronic data capture： | CRF & electronic data capture |
| Data Management Committee： | Not yet |

# Participants’ Statement

Consent Form

Research name: Efficacy and Safety of Anlotinib Combined with Penpulimab and XELOX Versus XELOX as Postoperative Adjuvant Treatment for ctDNA-positive Gastric/Gastroesophageal Junction Adenocarcinoma: A Protocol for a Randomized, Controlled, Multicenter Phase Ib Clinical Trial

Research number:

Research institute: Jiangsu Province Hospital, the First Affiliated Hospital with Nanjing Medical University.

Physician in charge of the study:

You are invited to participate in a clinical study, and this informed consent form provides some information to help you decide whether to participate in this clinical study. Please read it carefully. If you have any questions, please ask the researchers responsible for the study.

Your participation in this study is voluntary. This study was reviewed by the ethics review committee of the research institute. If you have questions related to the subjects' rights and interests, please contact the ethics committee of Jiangsu Province Hospital at +86-25-6830-6360.

Research purpose: This study will assess the response and side effects of ctDNA-guided reinforcement of anlotinib and penpulimab to standard XELOX treatment in patients undergoing D2 gastrectomy. We expect this regimen to have superior clinical efficacy and manageable side effects. The prognostic value of ctDNA, other hallmark molecules and nutritional status will also be explored.

Research process: If you agree to participate in this study, we will number each subject and create a medical record file. Due to the need for clinical diagnosis or treatment, you will have to undergo invasive testing or surgical operations, and the tissue will be removed for routine clinical pathological examination. Your case report will be published on websites and journals around the world. The printed and online versions will be available to doctors, media and the public.

Risk and discomfort: All information will be confidential. Your treatment shall be performed by medical professionals of various disciplinaries not limited to oncology, as the situation dictates.

Benefits: Precision diagnosis on a molecular basis, and part of the experimental regimen will be offered free of charge. Additional medical advice to your treatment shall be available based on that. By studying your case, we may provide necessary insight into the pathological nature of this disease and thus benefit all future patients.

Certain responsibilities that you shall take up as you agreed to participate in our study: You shall provide true information about your medical history and current physical condition; Inform the study physician of any discomfort during the study period; Not take restricted drugs, food, etc.; Tell your research doctor if you have been involved in other studies recently or are currently involved in other studies.

Privacy issue: If you decide to participate in this study, your personal data gathered in and beyond the study’s duration shall be confidential. Your tissue specimen will be identified by a study number, rather than your name. Information that could potentially lead to the disclosure of your identity will not be available to anyone other than members of the research group unless your permission is given. All research members and research bidders are required to keep your identity confidential. Your file will be kept in a locked filing cabinet accessible to researchers only. To ensure that the study is conducted in accordance with the regulations, if necessary, members of the government management department or the ethics review committee may refer to your personal data in the research unit as needed. When the results of this study are published, no information about you will be disclosed.

If you are injured by participating in this study, you can receive free treatment and/or compensation if you experience any harm associated with this clinical study.

You may choose to refuse to participate in this study. Even if you agreed now, you retain the right to request a full withdrawal of consent to the investigator. This will terminate your participation in this study and expel your data from the study results. Rest assured that your medical treatment and benefit would remain unaffected.

However, if additional treatment is needed, signs of any harm associated with this study are found, or intolerable deviation from the study plan is discovered, the investigator may also terminate your participation in this study.

You can keep track of the related information and the progress of the study. If you have any questions, experience any discomfort or injury during the study, or have any questions about the rights and interests of participants in this study, you can contact us via the ethics committee of Jiangsu Province Hospital, the First Affiliated Hospital with Nanjing Medical University at +86-25-6830-6360.
