# Supplementary material for: Efficacy and safety of XELOX combined with anlotinib and penpulimab vs XELOX as an adjuvant therapy for ctDNA-positive gastric and gastroesophageal junction adenocarcinoma: a protocol for a randomized, controlled, multicenter phase II clinical trial (EXPLORING study)
Source: Front Immunol. 2023 Oct 31;14:1232858. doi: 10.3389/fimmu.2023.1232858 (PMC10644233; doi:10.3389/fimmu.2023.1232858)
Supplement: Supplementary file 2 [file Table_1.docx]

Supplementary Material

**Efficacy and safety of XELOX combined with anlotinib and penpulimab vs XELOX as an adjuvant therapy for ctDNApositive gastric and gastroesophageal junction adenocarcinoma: a protocol for a randomized, controlled, multicenter phase II clinical trial (EXPLORING study)**

Yizhang Chen^1,2*^, Jiaguang Zhang^1*^, Gaohua Han^3*^, Jie Tang^4^, Fen Guo^5^, Wei Li^6^, Li Xie^7^, Hao Xu^8^, Xinyi Zhang^1^, Yitong Tian^1^, Lanlan Pan^1^, Yongqian Shu^1^, Ling Ma^1#^, Xiaofeng Chen^1,3#^

^1^: Department of Oncology, The First Affiliated Hospital of Nanjing Medical University, Nanjing, China

^2^: The Affiliated Wuxi People's Hospital of Nanjing Medical University, Wuxi People's Hospital, Wuxi Medical Center, Nanjing Medical University, Wuxi, China

^3^: Department of Oncology, The Affiliated Taizhou People's Hospital of Nanjing Medical University, Taizhou, China

^4^: Department of Oncology, Liyang People's Hospital, Changzhou, China

^5^: Department of Oncology, Suzhou Municipal Hospital, Suzhou, China

^6^: Department of Oncology, The First Affiliated Hospital of Soochow, Suzhow, China

^7^: Clinical Research Institute, Shanghai Jiao Tong University School of Medicine, Shanghai 200025, China.

^8^: Department of Gastric Surgery, the First Affiliated Hospital of Nanjing Medical University, Nanjing, China

*Yizhang Chen, Jiaguang Zhang and Gaohua Han contributed equally to this work.

**#Corresponding author:**

Ling Ma

Department of Oncology, The First Affiliated Hospital of Nanjing Medical University, No. 300,

Guangzhou Road, Nanjing, Jiangsu Province, China, 210029

maling@njmu.edu.cn

Xiaofeng Chen

Department of oncology, the First Affiliated Hospital of Nanjing Medical University, No. 300,

Guangzhou Road, Nanjing, Jiangsu Province, China, 210029

chenxiaofengnjmu@163.com

1. **Supplementary Table 1**

| Selected postoperative trials to explore prognostic value of ctDNA in gastric or gastroesophageal junction carcinoma | | | | |  |
| --- | --- | --- | --- | --- | --- |
| Study (finishing/starting year, depending on status) | Patient population | Number of participants (post-operative ctDNA positive vs. negative) | Timing of blood sample collection | Main outcome | Trial registration |
| Ueda et al(1). (2016) | Newly diagnosed esophageal squamous cell carcinoma (ESCC) | 4 vs. 0 | Before and after surgery | Allele frequencies (Afs) of all concordant mutations increased 9 months before recurrence was detected by imaging tests. In patients with nonrecurrence, somatic mutations derived from the primary tumor were also absent in serial plasma during the follow-up period. | N/A |
| Maron et al(2). (2019) | Adenocarcinoma of the esophagus, gastroesophageal junction, or stomach (GEA) | 7 vs. 15 | Prior to stage IV therapy initiation | Patients with detectable ctDNA (n=7/22) in samples drawn after curative-intent resection (median=50 days, range=20–135 days after surgery) had significantly diminished mDFS of 12.5 months versus unreached (P=0.03, HR=0.1, 95% CI 0.01–1.1) | N/A |
|  |  |  |  |  |  |
| Yang et al(3). (2020) | Stage I–III, resectable gastric cancer | 7 vs. 31 | Prior to any adjuvant chemotherapy; 9–48 days after surgery | ctDNA positivity after surgery was strongly associated with increased risk of relapse (100% recurrence in positive group vs. 32% in negative group, P = 0.0015, Fisher’s exact test) and worse DFS. | N/A |
| Aziz et al. (2016) | Localized or advanced adenocarcinoma of gastric or gastroesophageal junction | Estimated 200 in total | Parallel with chemotherapy sessions | N/A | NCT02674373 |
| Janjigian et al. (2020) | HER2+ esophageal, GEJ, or gastric adenocarcinoma undergone curative (R0) resection | Estimated 24 in total | 6 months postoperation | N/A | NCT04510285 |
| Mau-Sørensen et al. (2020) | Gastroesophageal cancer | Estimated 1950 in total | Over a period of 2 years | N/A | NCT04576858 |
| Aviano et al. (2022) | HER2-positive gastric cancer | Estimated 80 in total | Not mentioned | N/A | NCT05190445 |
| Shreenivas et al. (2022) | Locally advanced esophageal or gastroesophageal adenocarcinoma | Estimated 30 in total | Baseline, 14 days prior to surgery (approximately 8 months), 1 year, and 2 years | N/A | NCT05067842 |
|  |  |  |  |  |  |

1. Ueda M, Iguchi T, Masuda T, Nakahara Y, Hirata H, Uchi R, et al. Somatic mutations in plasma cell-free DNA are diagnostic markers for esophageal squamous cell carcinoma recurrence. Oncotarget. 2016;7(38):62280-91.

2. Maron SB, Chase LM, Lomnicki S, Kochanny S, Moore KL, Joshi SS, et al. Circulating Tumor DNA Sequencing Analysis of Gastroesophageal Adenocarcinoma. Clin Cancer Res. 2019;25(23):7098-112.

3. Yang J, Gong Y, Lam VK, Shi Y, Guan Y, Zhang Y, et al. Deep sequencing of circulating tumor DNA detects molecular residual disease and predicts recurrence in gastric cancer. Cell Death Dis. 2020;11(5):346.
